# Brazilian version of the postpartum depression screening scale-24

**DOI:** 10.1192/j.eurpsy.2021.1037

**Published:** 2021-08-13

**Authors:** M. Barros, M. Aguiar, A. Macedo, A.T. Pereira

**Affiliations:** 1 Departamento De Ciências Naturais, Universidade do Sudoeste da Bahia - UESB, Vitória da Conquista, Bahia, Brazil; 2 Instituto De Psicologia Médica, Universidade de Coimbra, Coimbra, Portugal; 3 Pos Graduação Em Psicologia Da Saúde, Universidade Federal da Bahia, Vitória da Conquista, Brazil; 4 Institute Of Psychological Medicine, Faculty Of Medicine, University of Coimbra, Coimbra, Portugal; 5 Institute Of Psychological Medicine, Faculty Of Medicine, University of Coimbra, coimbra, Portugal

**Keywords:** Reliability, Depression, Validity, Scale

## Abstract

**Introduction:**

The PDSS-24 is a Portuguese short version of the Postpartum Depression Screening Scale (Beck and Gable, 2002). Items were selected on the basis of exploratory factor analysis (those with loadings >.60). The PDSS-24 proved to be superior to the 35-items PDSS in reliability, validity and screening ability (Pereira et al. 2013).

**Objectives:**

To analyze the psychometric properties (construct validity using Confirmatory Factor Analysis, discriminant validity and reliability) of the Brazilian preliminary version of PDSS-24

**Methods:**

After confirming the items semantic equivalence and slightly adapt two adjectives from European to Brazilian Portuguese, 350 pregnant women (Mean age: 30.01±5.452; Mean gestation weeks=25.17±6.55), with uncomplicated pregnancies, completed the PDSS-24 and the Brazilian recently validated versions of Profile of Mood States-25 (PoMS; Barros et al. 2021). SPSS and AMOS software were used.

**Results:**

After some errors were correlated the multidimensional second-order model of PDSS-24 presented an aceptable fit (χ2=3.448; RMSEA=.099; CFI=.817, TLI=.886, GFI=.886). The PDSS Cronbach’s alpha for the total was α=.90. Cronbach alpha was .90 for the total and >.75 for the dimensions. Appling the Portuguese validated cut-off score for Major Depression/DSM-5 (>42) to this sample 224 (64.0%) participants presented clinical relevant depressive symptoms.

**Conclusions:**

The Brazilian PDSS-24 has acceptable validity and reliability. The percentage of women with high depressive symptomatology is three times higher than the figures reported in Portuguese Studies. This can be partly explained by the fact that data collection was done during the COVID19 pandemic. It is important to determine the PDSS cut-offs to screen for perinatal depression in Brazil.

